# *In Vitro* Dissolution and *In Vivo* Bioavailability of Six Brands of Ciprofloxacin Tablets Administered in Rabbits and Their Pharmacokinetic Modeling

**DOI:** 10.1155/2014/590848

**Published:** 2014-06-03

**Authors:** Sahar Fahmy, Eman Abu-Gharbieh

**Affiliations:** ^1^Department of Pharmaceutical Sciences, Faculty of Pharmacy, Helwan University, Cairo 11790, Egypt; ^2^Department of Pharmacology and Toxicology, Dubai Pharmacy College, P.O. Box 19099, Dubai, UAE

## Abstract

This study was undertaken to assess the *in vitro* dissolution and *in vivo* bioavailability of six brands of ciprofloxacin oral tablets available in the UAE market using rabbits. The *in vitro* dissolution profiles of the six ciprofloxacin products were determined using the USP dissolution paddle method. Pharmacokinetic modeling using compartmental and noncompartmental analysis was done to determine the pharmacokinetic parameters of ciprofloxacin after single-dose oral administration. *In vitro* release study revealed that the amount of ciprofloxacin released in 20 minutes was not less than 80% of the labeled amount which is in accordance with the pharmacopoeial requirements. All tested products are considered to be very rapid dissolving except for formulae A and D. Ciprofloxacin plasma concentration in rabbits was best fitted to a two-compartment open model. The lowest bioavailability was determined to be for product A (93.24%) while the highest bioavailability was determined to be for product E (108.01%). Postmarketing surveillance is very crucial to ensure product quality and eliminating substandard products to be distributed and, consequently, ensure better patient clinical outcome. The tested ciprofloxacin generic products distributed in the UAE market were proven to be of good quality and could be used interchangeably with the branded ciprofloxacin product.

## 1. Introduction

Introducing generic products from multiple sources into health care systems exist in many countries in an approach aiming to improving the overall healthcare system. However, this has been accompanied by a variety of problems, the most critical of which is the widespread distribution of counterfeit or substandard products.

Product selection of the same active ingredients from several generic products available in the market is very important step during the course of therapy and cause several concerns to a healthcare practitioner. Therapeutic equivalence must be insured by ascertaining the biopharmaceutical equivalency of such drug products [1].

Drug products that are therapeutically and chemically equivalent must have the same strength, quality, purity and content uniformity, and disintegration and dissolution rates [2]. The need to ensure that the generic and branded drug products are pharmaceutically and therapeutically equivalent cannot be overemphasized. Variable clinical response to the same dosage form of a drug product supplied by different manufacturers has been reported in literature [3, 4].

To reduce the medicines expenditure burden on a healthcare system, the World Health Organization (WHO) has continuously advocated the use of generic products but this should be supported with sufficient evidence for the substitution of one brand for another. This could not be achieved without proving its efficacy through bioequivalence studies [5].

Bioequivalence studies for generic products are essential to ensure the absence of any significant difference in the rate and extent to which the active ingredients become available at the site of drug action administered under similar conditions in an appropriately designed study [6].

However, evidences reported in literatures and in clinical practice as well indicate that marketed products with the same amount of active ingredient exhibit marked differences in their therapeutic responses [7]. This may be attributed to the in-similarity in the extent of drug absorption, due to different excipient used in the preparation of the different generic products. Bioequivalence studies focus on the release of drug from the formulation and subsequent absorption into the system's circulation. Bioequivalence studies may involve both* in vivo* and* in vitro* studies.

Dissolution testing, a surrogate marker for bioequivalence test, is a very practical and economic approach to identify bioavailability problems and assess the need for* in vivo* bioavailability [8]. Therefore, the* in vitro* dissolution is a vital tool in assessing the* in vivo* performance and also serves as a tool to identify unacceptable or substandard drug products.

The pharmaceutical market in the UAE is flooded with imported and locally manufactured generic products with no chemical/biopharmaceutical in-equivalencies have been reported so far. Raising this question, do these generic products have the same efficacy, quality, and safety? Quality control is the only answer to such question concerns of quality, safety, and efficacy of generic drugs in the market. Healthcare professionals are confronted with wide varieties of multisource generics and the only answer to their confusion will be performing quality control testing and bioequivalence testing for these products.

This study aims to assess the bioavailability of ciprofloxacin from selected generic products available in the UAE market. There are several brands of ciprofloxacin hydrochloride tablets available in the UAE market. The increasing use of ciprofloxacin hydrochloride tablets recently is a result of its versatility in the management of various cases of microbiological infections [9] and necessitated the need to evaluate the quality of the various available products.

Ciprofloxacin (CFX) is one from the fluoroquinolone groups which are synthetic broad spectrum bactericidal anti-infective agents with outstanding antibacterial activity against Gram-negative and certain Gram-positive bacteria as well as some Chlamydia and Mycoplasma, and many mycobacterium species [10–12].

Its mode of action is the inhibition of the essential enzyme for DNA replication and synthesis (DNA gyrase) [13]. It is approved for the treatment of 14 types of infections; the most common are urinary tract infections such as acute uncomplicated cystitis and chronic bacterial prostatitis, in addition to lower respiratory infections [14, 15]. Because of its potency, broad-spectrum activity, and general safety profile, ciprofloxacin is usually reserved to treat antibiotic-resistant infections [15].

For the health care providers to use these brands interchangeably, the bioequivalence of these brands have to be ascertained. This means that there should be continued postmarketing surveillance of the drugs. The purpose of this study is to study the bioavailability and the pharmacokinetic properties of ciprofloxacin after single-dose oral administration and to compare the absorption characteristics of the different generic products for ciprofloxacin compared to the branded product in rabbits.

## 2. Materials and Methods

All chemicals were analytical grade. Ciprofloxacin and lomefloxacin were obtained as a gift from Gulf Pharmaceutical Industries (RAK, UAE). The different ciprofloxacin brands A, B, C, D, E, and F were purchased from retail pharmacies in the UAE. Dissolution apparatus (Copley dissolution 6000, Copley Scientific, U.K.) using a paddle stirrer and an UV-1700 spectrophotometer (Shimadzu, Scientific Instrument Division) was used. Acetonitrile, acetic acid, HCl (0.1 N), and methanol were from Sigma-Aldrich. All solvents were of HPLC grade.

### 2.1. *In Vitro* Dissolution

The dissolution rates for all ciprofloxacin generic product as well as the reference product (Ciprobay) were tested (*n* = 6) using USP dissolution apparatus, paddle type (Copley, UK) maintained at 37 ± 0.5°C and rotation speed of 100 rpm. The dissolution media used were 1000 mL of 0.1 N HCl for one hour. Samples (5 mL) were withdrawn through syringe filter (0.8 micrometer) at predetermined time intervals. The withdrawn volume was replaced by the same volume of fresh dissolution media. Drug content was determined spectrophotometrically at 277 nm [15].

### 2.2. Analysis of* In Vitro* Dissolution Data

Difference factor (*f*_1_) and similarity factor (*f*_2_) were calculated to compare the dissolution profile of the different ciprofloxacin formulations [16]. Difference factor (*f*_1_) is used to measure the relative error between the two curves and is calculated as the percentage difference between two curves at each point. The similarity factor (*f*_2_) used to measure the similarity in the % of the drug dissolved between the two curves is calculated as the logarithmic reciprocal square root transformation of the sum of squared error. *f*_1_ and *f*_2_ factors are calculated using the following formulae.

The compared dissolution curves are considered similar and bioequivalent, if *f*_1_ is between 0 and 15 and *f*_2_ is between 50 and 100 [16]. Consider

(1) $$\begin{array}{c} f_{1}=\left\{\frac{\sum_{t=1}^{n}\left|R_{t}-T_{t}\right|}{\sum_{t=1}^{n}R_{t}}\right\},\\ f_{2}=50\log\left\{{\left[1+\frac{1}{n}\overset{n}{\underset{t}{\sum }}{\left(R_{t}-T_{t}\right)}^{2}\right]}^{-0.5}\right\}\cdot 100, \end{array}$$

where *n* is the number of time points, *R*_*t*_ is the dissolution value of the reference at time *t*, and *T*_*t*_ is the dissolution value of the test at time *t*. The *f*_2_ is basically a measurement of the similarity in the percent (%) drug dissolution between the two curves. Values of 50 or above (50–100) ensure similarity (difference ≤ 10%) of the curves.

### 2.3. Animals

Twenty-four healthy white albino rabbits of either sex ranging in body weight from 1 to 1.2 kg were used. All the animals were maintained under similar conditions. The animals were fed with fresh green fodder and black gram in the morning and evening, while water was provided freely as much they required.

### 2.4. Protocol of the Study

Pharmacokinetics of Ciprofloxacin from the different generic formulations was studied after administration of an oral dose in normal rabbits. The study was approved by the research and ethical committee in Dubai Pharmacy College.

### 2.5. Drug Administration

Rabbits were randomly divided into four groups (*n* = 6) and assigned to one of the selected drug preparations, that is, A, B, C, D, E, and the reference F. Tablets were crushed and mixed with carboxymethylcellulose (CMC) 1% w/v solution, ensuring that rabbits consumed all the dose. Drug was prepared in a solution form and was administered through the feeding tube orally. A single dose was given for each rabbit and was administered as a single dose of 250 mg/kg of body weight.

### 2.6. Sampling Procedure

The blood samples were collected through the central vein of the rabbits in heparinized glass centrifuge tubes with the aid of sterilized disposable plastic syringes just before and at 0.25, 0.5, 1, 1.5, 2, 4, 8, 12, and 24 hrs after the drug administration. The blood samples were centrifuged at 5000 rpm for 10 min to separate the plasma for analysis.

### 2.7. Drug Analysis

The concentration of CFX in plasma was determined by the high performance liquid chromatographic procedure as described by Kordick et al. with some modifications [17].

Analysis of samples was performed using HPLC system equipped with Waters 1515 pump, Waters 2487 detector, Waters 717 P auto-samplers, and Waters C18 column (5.0 *μ*m, 3.9 mm × 150 mm). The mobile phase used was a mixture of distilled water (81%) and acetonitrile (19%) and a small concentration of trifluoroacetic acid (0.02%) its flow rate was adjusted at 1.0 mL/min. The mobile phase was filtered and sparged with helium prior to use.

The ultraviolet (UV) detector was set at a wavelength of 279 nm. The column temperature was adjusted to be around 40°C in all cases.

The retention time for ciprofloxacin was 3.9 minutes with limit of detection (LOD) of 0.02 *μ*g/mL and limit of quantification (LOQ) of approximately 0.05 *μ*g/mL. Drug concentration was calculated by interpolating CFX peak areas on a calibration curve of spiked the blank plasma over the range assayed.

### 2.8. Sample Preparation

An equal amount of 5% perchloric acid was added to the plasma samples to separate proteins, vortexed for two minutes, and then centrifuged at 2000 rpm for 10 minutes. The aliquot was separated for injecting into the HPLC system.

### 2.9. Pharmacokinetic Analysis

Pharmacokinetic analysis was performed by both compartmental and noncompartmental approaches using WinNonlin PK software (Scientific Consulting Inc., Apex, NC). Plasma concentrations of ciprofloxacin versus time profile for selected generic formulation and the reference of each animal under nonsteady state condition were used to determine the disposition kinetic variables using compartmental model [18] and noncompartmental pharmacokinetic model based on the statistical moment theory [19]. Area under the plasma concentration-time curve (AUC) and the area under the moment curve (AUMC) were determined using trapezoidal method. Other pharmacokinetic parameters were derived using the following equations:

(2)  Mean  residence  time  (MRT)=AUMCAUC, Overall  elimination  rate  constant  (Kel)=1MRT, Biological  half-life  (t1/2)=0.693×MRT,Total  body  clearance  (Cl)=DoseAUC, Predicted  steady  state  plasma  concentration  (Css)of  drug  with  24 h  as  the  dosing  interval(t)=AUCt.

To compare ciprofloxacin bioavailability from the different formulae, relative bioavailability of the generic products was calculated using the following formula:

(3) $$\begin{array}{c} \text{Relative}\;\text{bioavailability}\;\left(F_{r}\right)=\frac{\left[{\text{AUC}}_{T}\right]\cdot D_{R}}{\left[{\text{AUC}}_{R}\right]\cdot D_{T}}, \end{array}$$

where *F*_*r*_ is the relative bioavailability in (%), AUC_*T*_ is the area under the curve for the test product, AUC_*R*_ is the area under the curve for the reference product, *D*_*R*_ is the dose of the reference, and *D*_*T*_ is the dose of the test product.

### 2.10. Statistical Analysis

Results are expressed as mean ± S.D. for triplicate samples. The statistically significant difference among the groups was determined by one-way analysis of variance (ANOVA) using SPSS^©^ statistical software (Version 16; SPSS Inc., Chicago, IL, USA). Statistical significance was considered at a level of *P* < 0.05.

The pharmacokinetic data derived by compartmental and noncompartmental methods were statistically compared applying Tukey's *A* test with *P* < 0.05 as the significant level of difference.

## 3. Results

### 3.1. Dissolution Testing

**Figure 1:**
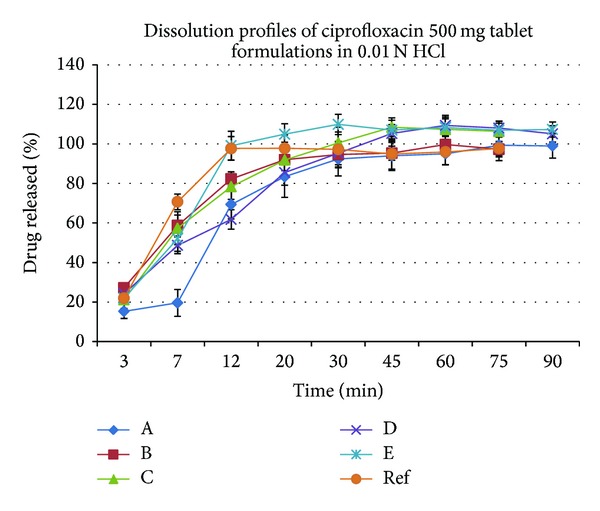
Dissolution profiles of ciprofloxacin (500 mg tablets) from the five tested generic products and the reference in 0.01 N HCl.

Figure 1 shows the dissolution profiles of the selected tablets of ciprofloxacin in 0.1 N HCl. In all cases, the amount of ciprofloxacin released in 20 minutes was not less than 80% of the labeled amount. This is in accordance with the pharmacopoeial requirements where it is stated that at least 80% of the ingredients are to be released within 30 minutes of dissolution. Most products may be considered as very rapidly dissolving as more than 85% of the labeled amounts of the drug substance dissolve within 15 minutes. The only exceptions were A and D.

These results suggest that the formulation and/or the manufacturing process can affect the dissolution and thus the bioavailability of the drug product. Proper drug formulation will allow for the drug to reach its site of absorption, the upper part of the GI tract (duodenum/jejunum) in a solution form. The bioavailability will then be determined by its* in vivo* permeability pattern. An* in vivo* bioequivalence study will establish whether the observed differences in the* in vitro* dissolution profile are significant* in vivo*.

Differences in the* in vitro* dissolution profiles among the studied generic products were assessed using the model-independent approach based on the similarity factor (*f*_2_). The calculated similarity factor for all formulations is presented in Table 1.

From the presented data, it is noted the difference factor for all formulations compared to the reference data was within the range of 0–15 and the dissolution profiles for all formulations were comparable to that of the reference with a similarity factor (*f*_2_) value greater than 50 except for formulae A and D. This indicates that these brands can be used interchangeably except for brand A and D.

**Figure 2:**
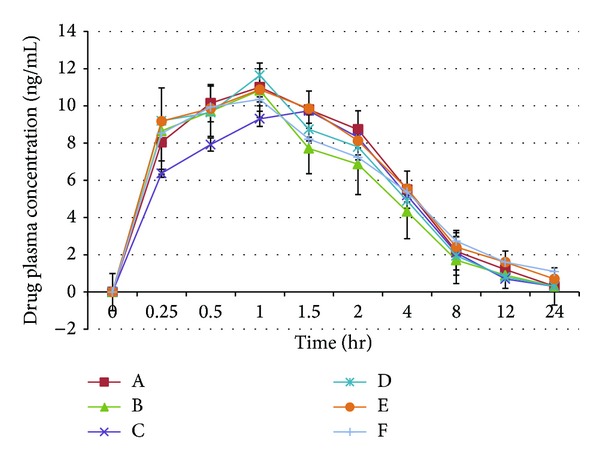
: Drug plasma concentration profiles after the administration of the 500 mg Ciprofloxacin dose from the five generic products and the reference in rabbits.

The mean drug plasma concentration after the administration of the five generic ciprofloxacin products as well as the reference product is shown in Figure 2. It is observed that all products reached maximum concentration after one hour of drug administration except for formula C where *C*_max⁡_ was reached after 1.5 hrs. The maximum drug concentration reached was 10.35 mg/mL for the reference products compared to 11.0, 10.83, 9.73, 11.64, 10.87, and 10.35 mg/mL from formulae A, B, C, D, and E, respectively.

**Figure 3:**
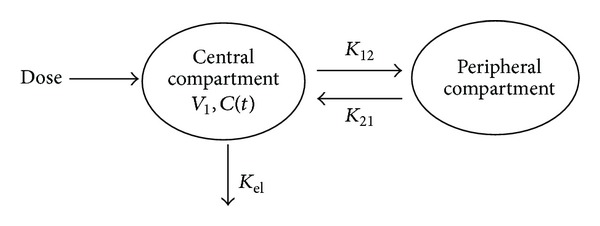
Two-compartment open model.

The plasma concentration-time data was best fitted to a biexponential equation corresponding to a two-compartment open model. The distribution (*α*) and elimination (*β*) phase regression lines were determined by the least square regression methods (Figure 3).

The noncompartmental and compartmental pharmacokinetic parameters (Mean ± SD) of ciprofloxacin after the oral administration of the different formulations in rabbits are presented in Tables 2 and 3, respectively.

The disposition kinetic parameters of ciprofloxacin based on the noncompartmental model of analysis are also listed in Table 2. The values of biological half-life, AUC, and MRT for ciprofloxacin were calculated within the range of 2.99–4.17 hr with the longest biological half-life for ciprofloxacin which was estimated to be 4.17 hr. The area under the curve and mean residence time were calculated to be within the range of 40.04–47.99 (hr∗mg/L) and 2.74–3.06 hr, respectively. The smallest volume of distribution was noticed for formula A with a value of 37.67 L and the largest volume of distribution was determined for the branded product with a value of 48.32 L.

Times to reach maximum drug concentration in plasma, maximum drug concentration, elimination rate constant, and renal clearance were not influenced by different generic products with a nonsignificant *P* value greater than 0.05. Although elimination half-life was prolonged for the reference (4.17 hr), this was not statistically different from other products. There was a statistically significant difference among the different formulations with respect to AUC and AUMC using multivariate analysis (ANOVA). Tukey's *A* test was performed and estimated that AUC last, AUC inf., and AUMC were significantly smaller for product B than other products.

Following compartmental model, the distribution and elimination half-lives of ciprofloxacin in rabbits were ranging between 0.34 and 3.63 hr and 2.99 and 4.32 hr, respectively. Time to reach maximum concentration was comparable with a maximum *T*_max⁡_ of 0.99 hr for formula C. *C*_max⁡_ was within the range of 9.57–10.89 mg/L.

Times to reach maximum drug concentration in plasma, maximum drug concentration, and renal clearance were not influenced by the different generic products with a nonsignificant *P* value greater than 0.05. However, elimination half-life was prolonged for the reference product (4.32 hr) as estimated from the noncompartmental analysis but found to be more slightly significant than other generic products.

There was a statistically significant difference among the different formulations with respect to the estimated AUC with product B of smaller area under the curve (46.60 ± 2.54 hr∗mg/L) compared to other products with a *P* value less than 0.05 (Tukey's *A* test).

### 3.2. Bioavailability

The relative bioavailability of the different generic products compared to the reference was calculated as shown in Table 4. The lowest bioavailability was shown to be for product A (93.24%) while the highest bioavailability was seen from product E (108.01%). Despite these results, it is evident that all generic products were of bioavailability greater than 90% concluding that these products could be used interchangeably with the reference product Ciprobay from Bayer.

## 4. Discussion

Six ciprofloxacin branded products were used and were within their shelf life as at the time of the study. The different brands of ciprofloxacin hydrochloride tablets were obtained from different retail pharmacy outlets within the UAE and were subjected to both* in vitro* dissolution and* in vivo* evaluation in rabbits to assess their bioavailability.

According to the FDA guidance for the industry concerning the dissolution testing of immediate release solid oral dosage forms, the biopharmaceutical classification system (BCS) suggests that drugs classified with class I and in some cases class III should release at least 85% of the drug upon drug dissolution in 0.1 N HCl in 15 min.; in this type, the bioavailability of the drug is not limited by dissolution [16]. Ciprofloxacin is a class III [20] drug and, from Figure 1, brands A, B, C, E, and F released ciprofloxacin greater than 85% at 15 min., which predicts that these formulations will have good bioavailability. The amounts of ciprofloxacin released by brands A and D were below 85% and reached 83.5% and 85.5% after twenty minutes, respectively.

To compare the dissolution profiles of the different generics versus the brand, a model independent approach of difference factor (*f*_1_) and similarity factor (*f*_2_) was employed. Similarity factor *f*_2_ has been adopted by FDA as a criterion to compare the similarity of two or more dissolution profiles. Similarity factor *f*_2_ is included by the Centre for Drug Evaluation and Research (CDER) in their guidelines such as guidance on dissolution testing of immediate release solid oral dosage forms [16] and guidance on waiver of* in vivo* bioavailability and bioequivalence studies for immediate-release solid oral dosage forms based on a biopharmaceutics classification system [21]. However, similarity factor *f*_2_ has some limitations in certain aspects of not taking into consideration the dissolution differences within the reference product and the test product batches [22]. In addition, it is reported by Costa to be insensitive to the shapes of dissolution profiles and does not put into consideration unequal spacing between sampling time points [23]. Despite these disadvantages, similarity factor *f*_2_ is represented to be a very simple and viable comparison approach to assess bioequivalence between the different formulations. In our study, the similarity factor *f*_2_ shows that generic products B, C, and E may be used interchangeably with the brand F from Bayer using the model independent approach.

In the present study, ciprofloxacin mean plasma concentrations in rabbits were determined and were between 9.57 and 10.89 mg/L, which are many times greater than the MICs for most susceptible organisms. The relative bioavailability of all ciprofloxacin formulations in rabbits was 93% to 108% after a 500 mg oral dose. Bioavailability was calculated by using AUC ratios corrected for dose. Following oral administration of the different ciprofloxacin formulations, ciprofloxacin plasma concentration versus time data in rabbits were best fitted to a two-compartment open model. This is comparable to the results of other studies in sheep [24], goats [25], and dogs [26].

The elimination half-life of ciprofloxacin after compartmental analysis in rabbits was determined to be within the range of 2.99–4.32 h and was longer than that of 2.44 h in preruminant calves [27] and 1.2 h in sheep and shorter than that of 4.85 h in horses [28], suggesting species-dependent differences in pharmacokinetic disposition of ciprofloxacin.

The apparent volume of distribution (*V*_*d*_) was determined by both compartmental and noncompartmental analysis and found that it is greater than 1.0 L/kg which suggests substantial tissue penetration ability of the drug. In addition, the high AUC value of ciprofloxacin determined in rabbit also emphasizes the high distribution of ciprofloxacin in body fluids and tissues and hence could be effectively used for the treatment of various systemic as well as deep seated infections [29].

Based on the compartmental model of analysis, distribution kinetic parameters, namely, distribution rate constant, distribution half-life, and rate constants of the transfer of drug from central to peripheral compartment (*K*_12_) and peripheral to central (*K*_21_), suggested rapid distribution of the drug from blood to tissues and body fluids. There were no significant differences between the values of certain pharmacokinetic parameters (elimination half-life, maximum drug concentration, renal clearance, and volume of distribution), suggesting that if compartmental model is applied without any bias, then it is as accurate, reliable, and effective as the model-independent analysis [29].

From both studies, the* in vitro* and the* in vivo*, it is proven that there are some variations among the generic products studied compared to the branded products. Despite that, it was proven that no generic product tested was considered as fake product. This is unlike other studies which reported that some of the tested ciprofloxacin generic products available in Nigeria were fake based on the results obtained for its quality control parameters. Other generic ciprofloxacin products were determined to be substandard products where their dissolution profile could not achieve 70% dissolution at 45 minutes or achieved 70% dissolution throughout the 1 hour period of the determination [30]. Another published study on 85 generic products available in 21 countries reported that 91% of the generic piroxicam products evaluated failed to meet the* in vitro* USP quality assurance criteria for potency and/or dissolution [31]. This difference in dissolution could result in altered bioavailability and hence potency, which may result in therapeutic failure. The current study demonstrated that the differences in the dissolution profiles and hence the bioavailability of the different brands of ciprofloxacin products available in the UAE market are demonstrated to be minimal.

## 5. Conclusion

The high demand for having the different generic products is essential to reduce the pharmaceutical expenditure worldwide. However, postmarketing monitoring is very crucial to ensure better clinical outcome. The problem of fake and substandard medicines remains the big challenge that regulatory authorities may face and thus arises the need for adequate quality assurance and quality control of drugs. Assessment of bioavailability of the different generic products available in the market is very important to ensure that generic drugs being sold can be used interchangeably with the branded products. In the UAE, all tested ciprofloxacin generic products were proven to be of good quality and there is no fake or substandard medicine identified to be marketed in the UAE market.

## Figures and Tables

**Table 1:** The calculated similarity factor and difference factor for all ciprofloxacin dissolution profiles obtained from the 5 tested generic ciprofloxacin products compared to the reference.

Product	*f*_2_	*f*_1_
A	44.63	13.55
B	66.95	3.56
C	58.17	0.78
D	48.40	3.73
E	58.43	5.90

**Table 2:** Noncompartmental analysis of ciprofloxacin from the tested products and the reference products after oral administration in rabbit.

Parameter	A	B	C	D	E	F
*T*_max⁡_ (hr)	1 ± 0	1 ± 0	1.5 ± 0	1 ± 0	1 ± 0	1 ± 0
*C*_max⁡_ (mg/L)	11 ± 0.12	10.83 ± 0.13	9.73 ± 0.16	11.64 ± 0.11	10.87 ± 0.11	10.35 ± 0.13
AUC last (hr·mg/L)	47.99 ± 0.35	40.04 ± 0.52*	44.23 ± 0.72	44.44 ± 0.62	47.97 ± 0.22	45.763 ± 0.49
Lambda_z (1/hr)	0.23 ± 0.01	0.23 ± 0.008	0.23 ± 0.015	0.23 ± 0.01	0.21 ± 0.01	0.177 ± 0.01
HL_Lambda_z (hr)	3.001 ± 0.19	2.99 ± 0.19	3.02 ± 0.195	3.00 ± 0.18	3.33 ± 0.17	4.17 ± 0.20
AUC INF_obs (hr·mg/L)	57.45 ± 2.07	47.42 ± 1.95*	53.46 ± 2.10	52.89 ± 1.54	59.58 ± 1.14	62.24 ± 1.98
Vz_F_obs (L)	37.67 ± 1.02	45.51 ± 0.95	40.76 ± 1.072	40.96 ± 1.24	40.31 ± 1.34	48.32 ± 0.89
Cl_F_obs (L/hr)	8.703 ± 0.21	10.54 ± 0.25	9.36 ± 0.37	9.45 ± 0.24	8.39 ± 0.16	8.04 ± 0.26
AUMClast (hr·hr·mg/L)	138.02 ± 3.12	109.93 ± 3.29*	130.55 ± 4.62	124.23 ± 3.65	140.69 ± 1.36	140.03 ± 3.25
MRT last (hr)	2.87 ± 0.04	2.74 ± 0.03	2.95 ± 0.06	2.79 ± 0.04	2.93 ± 0.03	3.06 ± 0.04

*P < 0.05.

**Table 3:** Compartmental analysis of ciprofloxacin from the tested products and the reference product after oral administration in rabbit.

Parameter	A	B	C	D	E	Ref
*A* (mg/L)	12.56 ± 0.12	17.73 ± 0.17	1.24 ± 0.09	1.05 ± 0.13	1.20 ± 0.11	22.46 ± 0.21
*B* (mg/L)	11.36 ± 0.05	10.44 ± 0.12	11.82 ± 0.08	12.44 ± 0.24	11.59 ± 0.07	9.94 ± 0.19*
Alpha (1/hr)	2.01 ± 0.04	2.03 ± 0.05	0.64 ± 0.15	0.24 ± 0.12	0.25 ± 0.06	2.22 ± 0.05
Beta (1/hr)	0.24 ± 0.005	0.23 ± 0.007	0.22 ± 0.003	0.23 ± 0.004	0.21 ± 0.006	0.16 ± 0.002*
K_12_ (1/hr)	0.57 ± 0.02	0.62 ± 0.03	0.01 ± 0.006	0.07 ± 0.003	0.01 ± 0.007	0.77 ± 0.102
K_21_ (1/hr)	1.16 ± 0.24	1.28 ± 0.32	0.63 ± 0.13	0.01 ± 0.003	0.24 ± 0.093	1.35 ± 0.052
AUC (hr·mg/L)	56.20 ± 1.85	46.60 ± 2.54*	52.92 ± 2.48	74.53 ± 3.57	61.15 ± 4.25	62.23 ± 3.27
Alpha_HL (hr)	0.37 ± 0.01	0.34 ± 0.05	1.84 ± 0.03	1.56 ± 0.06	3.63 ± 0.02	0.31 ± 0.04
Beta_HL (hr)	3.01 ± 0.25	3.07 ± 0.37	3.15 ± 0.29	2.99 ± 0.21	3.43 ± 0.035	4.32 ± 0.33*
V1_F (L)	29.20 ± 2.54	29.90 ± 2.96	41.73 ± 3.51	41.80 ± 3.21	40.62 ± 3.24	30.41 ± 3.75
CL_F (L/hr)	10.90 ± 0.34	10.70 ± 0.42	9.46 ± 0.63	6.70 ± 0.41	8.19 ± 0.95	8.04 ± 0.85
V2_F (L)	11.36 ± 0.23	14.50 ± 0.39	0.23 ± 0.03	10.35 ± 0.51	1.30 ± 0.24	17.33 ± 1.61
*T*_max⁡_ (hr)	0.54 ± 0.1	0.60 ± 0.3	0.99 ± 0.3	0.56 ± 0.2	0.61 ± 0.09	0.60 ± 0.07
*C*_max⁡_ (mg/L)	10.89 ± 0.95	10.57 ± 1.73	9.57 ± 0.93	10.49 ± 1.01	10.84 ± 0.57	10.45 ± 0.85

*P < 0.05.

**Table 4:** Relative bioavailability of the 5 tested generic products compared to the reference.

Bioavailability	A	B	C	D	E
(%)	93.24	98.05	104.01	101.58	108.01
